# The Microbial Rosetta Stone Database: A compilation of global and emerging infectious microorganisms and bioterrorist threat agents

**DOI:** 10.1186/1471-2180-5-19

**Published:** 2005-04-25

**Authors:** David J Ecker, Rangarajan Sampath, Paul Willett, Jacqueline R Wyatt, Vivek Samant, Christian Massire, Thomas A Hall, Kumar Hari, John A McNeil, Cornelia Büchen-Osmond, Bruce Budowle

**Affiliations:** 1 Ibis Therapeutics, a division of Isis Pharmaceuticals, 1891 Rutherford Rd., Carlsbad, CA 92008, USA; 2 J & L Scientific Editing, , 704 Frontage Rd., Sundance, WY 82729, USA; 3 International Committee on Taxonomy of Viruses, Department of Epidemiology, Mailman School of Public Health, Columbia University, 722 West 168th Street, New York, NY 10032, USA; 4 Laboratory Division, Federal Bureau of Investigation, Washington, DC 20535, USA

## Abstract

**Background:**

Thousands of different microorganisms affect the health, safety, and economic stability of populations. Many different medical and governmental organizations have created lists of the pathogenic microorganisms relevant to their missions; however, the nomenclature for biological agents on these lists and pathogens described in the literature is inexact. This ambiguity can be a significant block to effective communication among the diverse communities that must deal with epidemics or bioterrorist attacks.

**Results:**

We have developed a database known as the Microbial Rosetta Stone. The database relates microorganism names, taxonomic classifications, diseases, specific detection and treatment protocols, and relevant literature. The database structure facilitates linkage to public genomic databases. This paper focuses on the information in the database for pathogens that impact global public health, emerging infectious organisms, and bioterrorist threat agents.

**Conclusion:**

The Microbial Rosetta Stone is available at . The database provides public access to up-to-date taxonomic classifications of organisms that cause human diseases, improves the consistency of nomenclature in disease reporting, and provides useful links between different public genomic and public health databases.

## Background

Thousands of different microorganisms affect the health and safety of the world's populations of humans, animals, and plants. Infectious microorganisms include species of bacteria, viruses, fungi, and protozoa. Many different medical and governmental organizations have created lists of the pathogenic microorganisms most relevant to their missions. For example, the Centers for Disease Control and Prevention (CDC) maintains an ever-changing list of notifiable diseases, the National Institute of Allergy and Infectious Disease (NIAID) lists agents with potential for use in bioterrorist attacks, and the Department of Health and Human Services (HHS) maintains a list of critical human pathogens. Unfortunately, the nomenclature for biological agents on these lists and pathogens described in the literature is imprecise. Organisms are often referred to using common names, alternative spellings, or out-dated or alternative names. Sometimes a disease rather than a particular organism is mentioned, and often there may be multiple organisms or co-infections capable of causing a particular disease. Not surprisingly, this ambiguity poses a significant hurdle to communication among the diverse communities that must deal with epidemics or bioterrorist attacks.

To facilitate comprehensive access to information on disease-causing organisms and toxins, we have developed a database known as "The Microbial Rosetta Stone" that uses a new data model and novel computational tools to manage microbiological data [1,2]. This article focuses on the information in the database for pathogens that impact global public health, emerging infectious organisms, and bioterrorist threat agents. It provides a compilation of lists, taken from the database, of important and/or regulated biological agents from a number of agencies including HHS, the United States Department of Agriculture (USDA), the CDC, the World Health Organization (WHO), the NIAID, and other sources. We curated these lists to include organism names that are consistent with the National Center for Biotechnology Information (NCBI) nomenclature and to provide sequence accession numbers for genomic sequencing projects (if available). Important synonyms or previously used names that identify the organisms are also shown. We have organized the lists according to phylogenetic structure. This paper provides graphic representations of the phylogenetic relatedness of important pathogenic organisms.

The goal of the database is to provide an informative, readily accessible, single location for basic information on a broad range of important disease causing agents. The database will help users to avoid the pitfalls of confusing nomenclature and taxonomic relationships and allow access to literature on in-depth studies. The database can be accessed at .

## Results

The Microbial Rosetta Stone database was designed to link genomic sequence data, taxonomic classification, and epidemiological information in a way that facilitates searches and data updates. The intent is not to replace currently available tools, like those available from NCBI, but instead serve to resolve ambiguities and data conflicts. One of the main goals of building this informatics infrastructure was to support the rapid determination of the causative agent in the event of a bioterrorist action or emergence of a previously unknown infectious disease.

This paper focuses on pathogenic organisms from several broad categories. Those currently known to impact global human health are monitored by a number of public health organizations and United States governmental agencies including the WHO [see Additional File 1] and the CDC [see Additional File 2]. Important food and water borne pathogens are listed in Additional File 3 [see Additional File 3]. Those agents responsible for emerging diseases are listed in Additional File 4 [see Additional File 4]. Pathogens considered high priority by the NIAID are listed in Additional File 5 [see Additional File 5]. Agents are listed that pose a high threat for bioterrorism [see Additional File 6], biocrimes [see Additional File 7], or have a high potential to be genetically manipulated [see Additional File 8]. Human pathogens formally regulated by the HHS are listed in Additional File 9 [see Additional File 9] and animal pathogens monitored by the U.S. Department of Agriculture (USDA) are listed in Additional File 10 [see Additional File 10].

**Figure 1:**
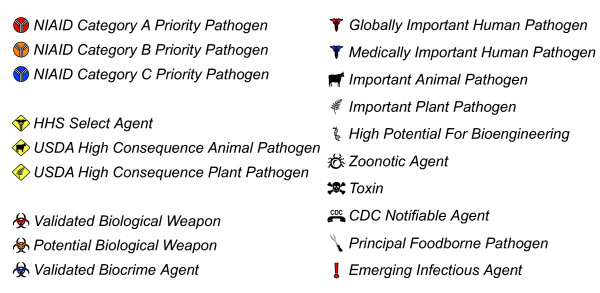
**Symbol key**. These symbols are used in the Figures 2 through 7 to link pathogen names to their originating Additional Files.

**Figure 2:**
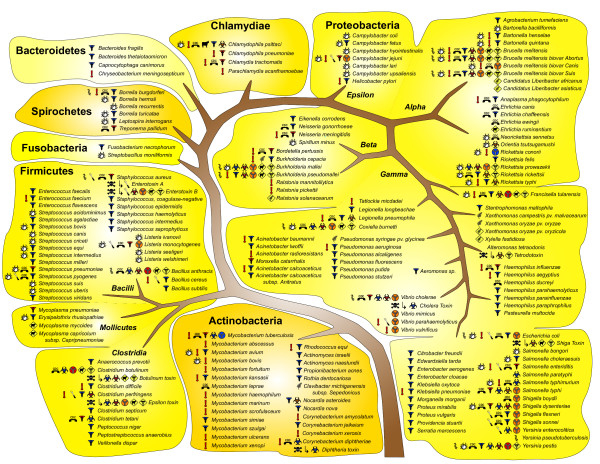
**Bacterial pathogens**. Bacterial phylogeny is based on work by Hugenholtz *et al. *[69]. Bacterial phyla are shown on the leaves, with the Firmicutes and the Proteobacteria subdivided to the class level (taxa are shown in italics). Their phylogeny is further broken down to the order level with the use of smaller branches. These two phyla account for roughly three quarters of the described infectious bacterial species (with the noticeable exception of deltaproteobacteria pathogens). Proteobacteria contain both important plant and animal pathogens. Proteobacterial plant pathogens are not clustered, but are observed in alpha, beta, and gamma subdivisions. Some plant and animal pathogen species share the same genus classification (e.g. *Ralstonia *and *Pseudomonas*), and for at least one species, *Burkholderia cepacia*, different subspecies are plant and human pathogens. Of the 20 bacterial phyla currently recognized in the NCBI taxonomy, thirteen are not present in Figure 2 due to the absence of noteworthy pathogens. Some of these missing phyla are relatively important, like the Cyanobacteria, but most of the remaining phyla are restricted to a handful of species and/or environmental niches. Also of interest is the relative weight of the infectious agents within their respective phylum: While virtually all Spirochaetes and Chlamydiae constitute potential infectious agents due to their parasitic lifestyle, the phylum Actinobacteria has few pathogens relative to the overall diversity of the phylum. It should be cautioned that our current view of bacterial diversity is biased towards cultivatable organisms, which represent a small fraction of bacterial diversity [69]. Therefore, this compilation represents the *well-recognized *infectious bacterial agents, but should in no way be considered exhaustive or complete.

**Figure 3:**
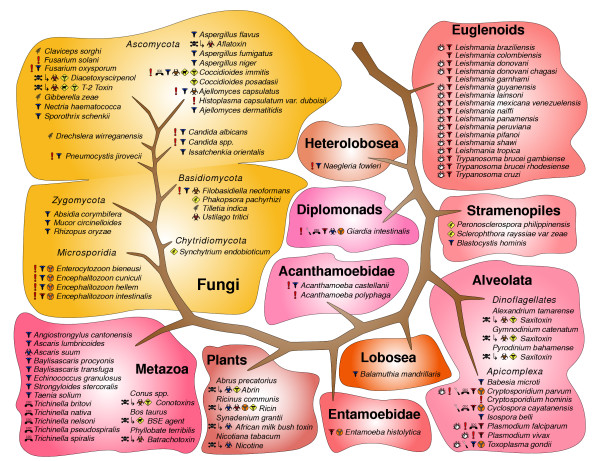
**Eukaryotic pathogens**. Eukaryotic pathogen life forms are clearly dominated by the fungi and protists. Within the Fungi, the phylum Ascomycota has many human, animal, and plant pathogens and is a major source of toxins. Protist species are responsible for globally important diseases such as the malaria-causing *Plasmodium *species and the *Leishmania *and *Trypanosoma *species that cause significant mortality in the developing world. The eukaryotic phylogeny tree is based on ribosomal and housekeeping gene sequence analysis [70], but different taxonomy levels are simultaneously represented as the topology has not yet been reliably determined.

**Figure 4:**
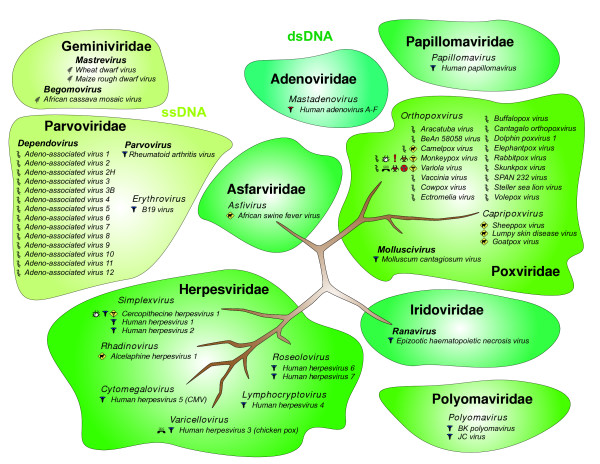
**DNA viruses**. The relationships for large genome DNA viruses on this chart was derived from the work of Iyer, Aravind & Koonin, who showed the common ancestry of four large DNA virus families [72]. Noteworthy in this figure are the number of viruses that may be readily bioengineered.

**Figure 5:**
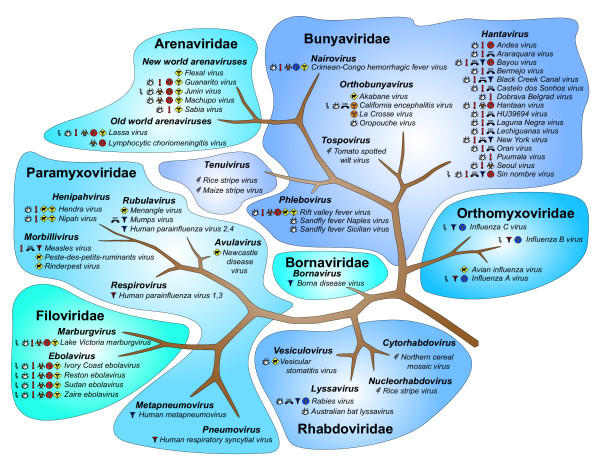
**Single-stranded negative strand RNA viruses**. The common origin of RNA viruses and their tentative relationships as indicated on this chart are based on an extensive analysis of their RNA-dependent RNA- or DNA-polymerases (C.M. manuscript in preparation). The branching of double-stranded RNA viruses is unresolved in light of their apparent polyphyly [73]. Virtually all known branches of ssRNA(-) viruses harbor pathogens and thus are represented in this panel. Only the rhabdoviridae family harbors both animal and plant pathogens. Particularly noteworthy are the deadly Ebola and Influenza viruses, the latter has claimed tens of millions of human lives in the past century.

**Figure 6:**
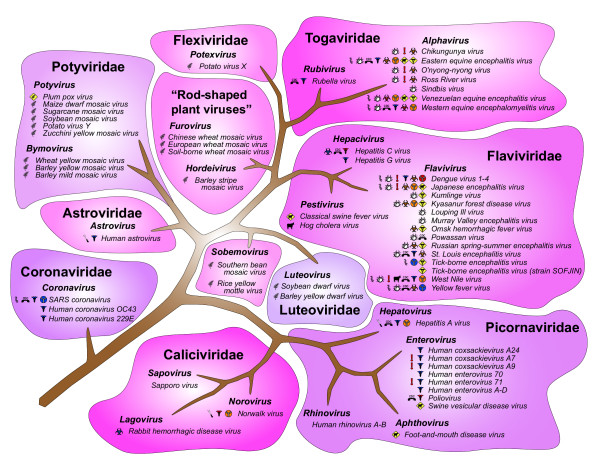
**Single-stranded positive strand RNA viruses**. The *Togaviridae *and *Flaviviridae*, and to a lesser extent the *Coronaviridae *and *Picornaviridae*, families are the most prominent human pathogens. Important plant viruses affect major cereal grains causing severe crop damages worldwide and affecting the nutrition of entire populations.

**Figure 7:**
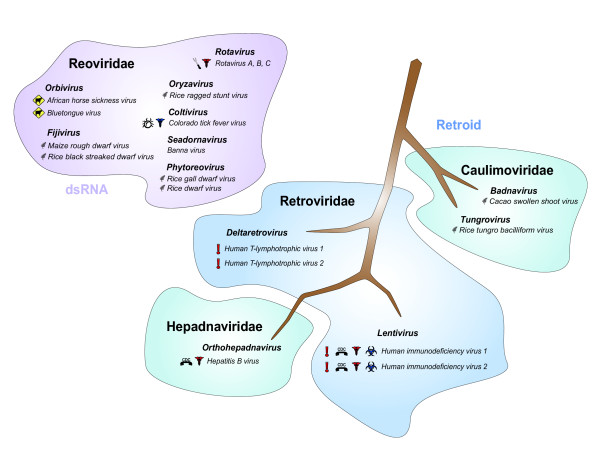
**Retroid viruses and double-stranded RNA viruses**. The most prominent include the two HIV viruses and the Hepatitis B virus, and the main family of double-stranded RNA viruses, the Orbiviridae. The latter family is an example of a virus family that includes both animal and plant pathogens. The plant pathogens primarily infect rice cultures.

The organisms listed in the Additional Files are represented according to phylogenetic hierarchy in the figures. The symbols used in the figures connect the organism names back to one or more of the Additional Files, which allows visualization of the fact that organisms are often represented in multiple Additional Files. Figure 1 is the symbols key. Figures 2 and 3 show the phylogenetic relationships of cellular life forms including bacteria, fungi, protozoa, and the plants and animals that produce toxins, Figure 4 shows the phylogenetic relationships of DNA viruses, and Figures 5, 6, and 7 show the phylogenetic relationships of RNA viruses.

For optimal utility, the genomic sequence data, taxonomic, and epidemiological information were linked in a way that facilitates searches, data updates, and data curation. The Microbial Rosetta Stone system will serve as a translator to resolve ambiguities and data conflicts between other public databases and literature and, when unable to resolve ambiguities, provide enough context for users understand the nature of the ambiguities. The database provides links to literature on experimental protocols for pathogen identification and to the genomic and biochemical information specific to a species, strain, or isolate. The data model and query tools are described in detail in Hari et al. [1]. The following sections describe the information and literature sources used to populate in the database for globally important and emerging pathogens and potential bioterrorist agents.

### Important public health pathogens

In the developing world, nearly 90% of infectious disease deaths are due to six diseases or disease processes: acute respiratory infections, diarrhea, tuberculosis, HIV, measles, and malaria [see Additional File 1] [3]. In both developing and developed nations, the leading cause of death by a wide margin is acute respiratory disease [3-5]. In the developing world, acute respiratory infections are attributed primarily to six bacteria: *Bordetella pertussis*, *Streptococcus pneumonia*, *Haemophilus influenzae*, *Staphylococcus aureus*, *Mycoplasma pneumonia*, *Chlamydophila pneumonia*, and *Chlamydia trachomati*s [6]. These bacteria belong to four different taxonomic classes and illustrate how similar parasitic lifestyles can evolve in parallel within unrelated bacterial species (Figure 2). Major viral causes of respiratory infections include respiratory syncytial virus (Figure 5), human parainfluenza viruses 1 and 3 (Figure 5), influenza viruses A and B (Figure 5), as well as some adenoviruses (Figure 4) [5,6].

The major causes of diarrhoeal disease in the developing and developed world have significant differences due to the great disparity of availability of pure food and water and the general nutritional and health status of the populations. Important causes of diarrhoeal disease in the developing world are those that tend to be epidemic, especially *Vibrio cholera*, *Shigella dysenteriae*, and *Salmonella typhi *[7]. These organisms are gammaproteobacteria (Figure 2) that use many different metabolic pathways to ensure their survival in a wide range of environments. In the United States there is a much lower incidence of diarrhoeal disease overall, and a relatively greater impact of direct human-to-human infectious transmission. The most important causes of diarrhoeal disease in the United States are bacteria such as *Escherichia coli*, *Campylobacter species*, *Clostridium difficile*, *Listeria monocytogenes*, *Salmonella enteritidis*, and *Shigella *species (Figure 2); viruses, such as Norwalk virus (Figure 6) and rotaviruses (Figure 7); and parasites such as *Cryptosporidium parvum*, *Cyclospora cayetanensis*, *Entamoeba histolytica*, *Giardia lamblia*, while microsporidia are responsible for a smaller number of cases (Figure 3).

Infectious disease agents important to the public health in the U.S. are monitored by the CDC and listed in Additional File 2 [see Additional File 2]. There are no set criteria for inclusion on the notifiable disease list; rather, the list is created by the CDC in cooperation with state health departments. As diseases occur less frequently and new diseases emerge, the notifiable disease list changes. The list provides links to case definitions of each disease, including the etiological agent(s) responsible. In cases where the etiological agent was not listed or was unspecific (i.e. *Brucella *spp.), further research was done to determine an etiological agent and this information is in Additional File 2 [see Additional File 2].

### Food-borne pathogens

Each year in the United States, there are approximately 76 million cases of food-borne illness, including 325,000 hospitalizations and 5,000 deaths [8,9]. In an estimated 2 to 3% of these cases, chronic sequelae develop. These sequelae include renal disease, cardiovascular diseases, gastrointestinal disorders, neural disorders, and autoimmune disease [10]. The estimated cost of food-borne illness in the United States is $23 billion annually [8]. Mishandling of food is believed to be responsible for 85% of all outbreaks of food-borne disease in developed nations, primarily due to a lack of education [8,11-13]. Food-borne pathogens [see Additional File 3] are also important because they represent one of the largest sources of emerging antibiotic-resistant pathogens. This is due in part to the administration of sub-therapeutic doses of antibiotics to food-producing animals to enhance growth. For example, certain strains of *Salmonella *show resistance to eight or more antibiotics [14]. Studies have shown that antibiotic resistance in *Salmonella *cannot be traced to antibiotic use in humans, suggesting that antibiotic use in animals is the primary cause of resistance [15].

While much is known about the major microbes responsible for diseases, there are still many undiagnosed cases of infectious disease. It has been estimated that as many as three-fifths of the deaths from acute gastroenteritis per year in the United States are caused by an infectious organism of unknown etiology [16]. Four of the major causes of food-borne infections (*Campylobacter jejuni*, *Escherichia coli *O157:H7, *Listeria monocytogenes*, and *Cyclospora cayetanensis*, Figure 2) were only recently recognized as causes of food-borne illness [16].

### Emerging infectious disease

Diseases that have recently appeared or that are growing in incidence are classified as emerging infectious diseases [see Additional File 4]. Emerging infectious organisms often encounter hosts with no prior exposure and thus represent a novel challenge to the host's immune system. Morse [17] identified six general factors in emergence of infectious disease: ecological changes, human demographics and behavior, international travel, technology and industry, microbial adaptation and change, and breakdown in public health measures. A comprehensive review by Taylor, Latham, and Woolhouse [18] identified zoonotic status as one of the strongest risk factors for disease emergence. Roughly 75% of emerging pathogens are zoonotic, and zoonoses are twice as likely to be considered emerging as non-zoonoses.

Zoonoses are a special class of pathogens as they co-evolve with the reservoir host, not with humans. Although not always the case, when zoonotic pathogens infect humans they have a tendency to cause severe disease, much like a commensal microorganism that infects unusual areas of the body [19]. Zoonoses can be broken down into two basic groups: those spread by direct contact with the infected animal and those spread via an intermediate vector [20]. Zoonoses can infect humans through many vectors. Members of the genus *Hantavirus *are spread by rats [21], West Nile virus by mosquitoes [22], and *Campylobacter *species by family pets [23]. In 2003, there was an outbreak of monkeypox (Figure 4) in the Midwestern United States, which was attributed to the importation of pet animals [24]. Viruses, protozoa, and bacteria can all be transmitted either directly or via a vector. However, these groups are clearly differentiated on a phylogenetic level. All zoonotic members of the families *Flaviviridae *and *Togaviridae *(Figure 6) are transmitted via an intermediate vector, while all zoonotic members of the families *Arenaviridae*, *Paramyxoviridae*, and *Filoviridae *(Figure 5) are transmitted through direct contact. Almost all zoonotic proteobacteria are transmitted through direct contact as well [20]. Of all the agents infectious to humans, a majority are zoonotic in origin [18].

Several viruses responsible for human epidemics have made a transition from animal host to human host and are now transmitted from human to human. Human immunodeficiency virus (Figure 7), responsible for the AIDS epidemic, is one example [25]. Although it has yet to be proven, it is suspected that severe acute respiratory syndrome (SARS), caused by the SARS coronavirus (Figure 6), also resulted from a species jump [26]. For many years, Robert G. Webster has studied the importance of influenza viruses in wild birds as a major reservoir of influenza viruses and has clarified their role in the evolution of pandemic strains that infect humans and lower animals [27].

Intriguingly, it appears that whenever a virus (or any other pathogen or pest) is eradicated another appears to fill the environmental niche. For example, in regions where the wild-type poliovirus (Figure 6) has been eliminated, non-polio enteroviruses have been associated with outbreaks of paralytic disease that clinically exhibit symptoms of poliomyelitis. The most common non-polio enteroviral causes of Acute Flaccid Paralysis (AFP) are human coxsackieviruses A7 and A9 and human enterovirus 71 (Figure 6). The poliovirus epidemics that emerged in the mid 19th century are now believed to have arisen not due to a change in the properties of the virus, but due to an improvement of public sanitation and personal hygiene, which delayed the acquisition of enteric virus infection from infancy to childhood. Since maternal antibodies protected infants, they usually underwent silent immunizing infections, whereas when older children were infected they more often suffered paralytic poliomyelitis [28].

### Economically important plant and animal pathogens

Proteobacteria are important plant and animal pathogens, and it is interesting that proteobacterial plant pathogens are not clustered on the phylogenetic tree, but are observed in alpha, beta, and gamma subdivisions (Figure 2). Some plant and animal pathogen species share the same genus classification (e.g. *Ralstonia *and *Pseudomonas*), and for at least one species, *Burkholderia cepacia*, different subspecies are plant and human pathogens. In viruses, plant and animal pathogens are generally found in distinct families, with the notable exception of the families *Rhabdoviridae *(Figure 5) and *Reoviridae *(Figure 7) that harbor both. Most plant-specific virus families are not shown in Figures 4,5,6,7, but for rice alone at least ten unrelated virus species have been reported to infect cultures worldwide. The overall economical effect of plant pathogens has been estimated to reduce crop production by one fifth [29]. Foot-and-mouth disease almost destroyed the beef industry in the United Kingdom and losses were very high. Bird flu in South East Asia affected the poultry industry severely. Influenza A is a bird virus that jumped from birds via pigs to humans and as a human pathogen has a large impact on the economy [30].

### Bioterrorism and biocrimes

Bioterrorism can be defined as an attack or threat of an attack using bioweapons on humans and/or their assets to create fear, to intimidate, to inflict harm, and/or affect economic well being. Such acts may be motivated by political, religious, ecological, or other ideological objectives. Any microorganism or toxin capable of causing disease or harm in humans, plants, or animals has the potential for illicit use and thus the list of potential agents could be vast. However, not all organisms or toxins make useful biological weapons. Displaying biological weapons from a phylogenetic perspective provides insight into how organisms that have been historically used as biowarfare agents are related to important human and agricultural pathogens and also provides insight into other similar organisms that might be considered as weapons in the future. The properties that make organisms amenable for use as biological weapons have been discussed extensively [31-39]. The most important features include: 1) accessibility; 2) consistent ability to cause death or disability; 3) culturability; 4) possibility for large scale production; 5) stability and ability to retain potency during transport and storage; 6) delivery potential; 7) stability and retention of potency after dissemination; and 8) infectivity and toxicity [39].

The infectious agents considered by the NIAID to have high potential for bioterror use are listed in Additional File 5 [see Additional File 5]. Validated and potential bioweapons agents are listed in Additional File 6 [see Additional File 6]. Agents that have been used to commit biocrimes [2] are in Additional File 7 [see Additional File 7]. The pathogens regulated by the HHS and the USDA are shown in Additional File 9 [see Additional File 9] and Additional file 10 [see Additional File 10], respectively. In addition to infectious microbes, toxins derived from biological sources are contained on these lists. We identified the biological source of each toxin, and listed the source organism in the Additional File containing the toxin.

All 11 regulated toxins can be found on the HHS Select Agent list [see Additional File 9] and five are also regulated by the USDA [see Additional File 10]. The regulated toxins (four small molecules and seven peptides or proteins) are produced by a wide variety of organisms: five are produced by bacteria [40-42], two by fungi [43,44], two by plants [45,46], one by an animal [47], and one by a protist [48]. The bacteria that produce toxins are indicated in Figure 2 and the eukaryotic toxin producers are shown in Figure 3. The conotoxins are a collection of five families of related peptide toxins produced by the various species of snails in the genus *Conus *[47]. The two fungal toxins, diacetoxyscirpenol and T-2 toxin, are produced by multiple species of the genus *Fusarium *[43,44]. Of the five regulated bacterial toxins, four are peptides or proteins and tetrodotoxin [49] is a small molecule. Tetrodotoxin is produced by a variety of gammaproteobacteria that colonize the puffer fish [50]. These toxins are relevant both due to concern that they may cause outbreaks of disease through accidental food contamination and because they are potential bioweapons. According to the CDC [51], an average of 110 cases of botulism, caused by a bacterial toxin (Figure 2), are reported each year. Interestingly, numbers of cases of food-borne and infant botulism have not changed significantly in recent years, but instances of wound-botulism have increased primarily due to the use of black-tar heroin, especially in California. Ricin is a toxin produced by the plant *Ricinus communis *(Figure 3) [46]. When prepared as a weapon, there are three methods of delivery: ingestion, injection, or inhalation [41]. One of the most famous documented uses of ricin was in the assassination of Georgi Markov, a Bulgarian defector who died three days after being shot with a ricin pellet [41,52].

### Organisms with high potential for genetic engineering

Pathogens that can be genetically manipulated [see Additional File 8] represent a unique bioterrorist threat, particularly as technology to genetically alter microbes becomes more commonplace. For the purposes of this work, viruses considered as having a high potential danger for bioterrorist engineering were derived from the literature reports and from the authors' judgment balancing the considerations of terror potential with the technical difficulty of use.

DNA viruses (Figure 4) with large genomes can be manipulated in culture, and are susceptible to engineering events such as insertion of genes not normally present in viral genomes. For example, the entire orthopoxvirus genus of the *poxvirus *family is considered to have high potential for bioengineering because of a much-publicized paper that describes the engineering of an orthopox member, ectromelia virus, to express mouse interleukin-4, resulting in a virus with extraordinary virulence [53]. Another member of the orthopoxvirus genus, vaccinia virus, is commonly used in molecular biology research and is extensively used in vaccines and vaccine research [54-57]. Thus, because they are highly infectious, will tolerate inserted genes, and can be manipulated easily in culture, poxviruses must be considered high-risk agents for acts of bioengineering. Mutations to create vaccine- and drug-resistant strains are also possible in other large-genome DNA viruses. Adeno-associated viruses are small DNA viruses that are members of the family *Parvoviridae*. They are satellite viruses that depend upon the presence of their helper DNA viruses, including members of the families *Adenoviridae *and *Herpesviridae*, for replication. Like vaccinia virus, adeno-associated viruses have been used extensively as vectors for gene therapy, and therefore represent a risk for genetic modification and subsequent terrorist use.

RNA-genome viruses are naturally more resistant to insertion of genes, but can be made resistant to drugs and potentially even to vaccines by introduction of mutations. Most critically, RNA viruses may be created in the laboratory by total chemical synthesis to generate active virus particles or reconstituted from cloned DNA. Total chemical synthesis was demonstrated recently for poliovirus (Figure 6) [58]. Since this publication in 2002, the technology to chemically synthesize viruses has advanced substantially. Chemical synthesis of RNA viruses would eliminate the need for the bioterrorist to obtain stock cultures and would allow creation of any wild-type or mutant strain of virtually any virus. Other positive-strand RNA viruses (Figure 6) that have been reconstituted from cloned DNA samples include Kunjin virus [59], human rhinovirus [60], foot-and-mouth disease virus [61], transmissible gastroenteritis virus, a coronavirus [62,63], all three genera of the family *Flaviviridae *[64], and a variety of plant and insect viruses [65,66]. Thus, the positive-stranded viruses must be considered a high-risk area for bioengineering, where the relative risk for each virus must be considered roughly equivalent to the terrorist potential inherent to the virus.

Negative-strand RNA viruses (Figure 5) can also be reconstituted by total chemical synthesis or from cloned genomic DNA by a reverse genetics approach [67]. Many negative-strand RNA viruses, including ebolaviruses, Lake Victoria marburgvirus, Hantaan virus and Lassa virus, are important human pathogens and potential bioterrorist agents. Influenza virus A is a particularly noteworthy negative-strand RNA virus due to its potential to cause pandemic disease, the availability of stock cultures of many strains, and the potential for total chemical synthesis by reverse genetics techniques [68].

For bacteria, if virulence factors have previously been modified successfully in a species, that species was considered to have a high potential for bioengineering and was included on the chart. However, numerous other methods for engineering bacterial genomes exist. Bacterial genomes consist of one or more chromosomes and additional genetic information may exist in the form of plasmids. Plasmids replicate independently of the chromosomal DNA and can be transferred between bacteria through transformation. Genes encoding virulence factors may be found in either chromosomes or plasmids. In one example of an early bioengineering event, the selective growth of an antibiotic-sensitive species with an antibiotic-resistant species in the presence of the antibiotic resulted in transference of resistance. Current techniques allow for the splicing of genes encoding virulence factors from one species followed by insertion into another. However, the complexity of the bacterial genome makes it difficult to successfully transfer a gene with a high rate of expression.

## Conclusion

This manuscript provides a visualization of the results obtained from use of the Microbial Rosetta Stone Database, a database that uses a new data model and novel computational tools to synthesize information on important human, animal, and plant pathogens [1]. Information on critical pathogens and diseases has been collected from many sources. Organism names from all lists were converted to NCBI species names and diseases were linked to particular pathogens, which facilitated computational analysis and linkage to public genomic databases. This database should facilitate access to information on disease-causing organisms and toxins. The database can be accessed at .

## Methods

Agent lists were collected from various sources as described in more detail for each Additional File below. Government agency lists (Additional Files 1, 2, 5, 9, and 10) were taken directly from the specified agencies, while additional tables were compiled using cited literature. Organism names from all lists were converted to NCBI species names. This included changes from species synonyms to NCBI-accepted nomenclature and expansion from *Genus *to *Genus species *(*e.g. Shigella *to *Shigella dysenteriae *or *Shigella sonnei*) to represent the most important species (when known). Where possible, disease names were associated with the name of the most predominant organism that causes the disease. The Additional Files contain taxonomic information, the NCBI name of the agent and any well-known synonyms, and accession numbers for the complete genome of each agent (if available).

Names of the infectious agents listed in the Additional Files were placed in tree-structure drawings shown in the figures that display the biological relatedness of the organisms. Cellular life forms were organized according to currently accepted phylogenetic relationships. Bacterial phylogeny (Figure 2) is based on work by Hugenholtz *et al. *[69]. The eukaryotic phylogeny tree (Figure 3) is based on ribosomal and housekeeping gene sequence analysis [70]. The branching order presented in Figure 3 should be considered tentative as the on-going recognition of additional protist lineages is likely to alter the topology of the eukaryotic tree in the near future [71]. The phylogeny of DNA viruses (Figure 4) was derived primarily from the work of Iyer, Aravind & Koonin [72]. The common origin of RNA viruses and their tentative relationships (Figures 5,6,7) is based on an extensive analysis of their RNA-dependent RNA- or DNA-polymerase (manuscript in preparation). The RNA virus phylogenetic trees were created using maximum parsimony on protein sequences. The branching of double-stranded RNA viruses is for now left unresolved in light of their apparent polyphyly [73]. The symbol key is shown in Figure 1 and symbols are used in the other figures to link the pathogens in the figures to Additional Files.

The agents listed in Additional File 1 [see Additional File 1] were taken from a list maintained by the WHO. The WHO tracks the occurrence of morbidity and mortality of global infectious diseases. Because of the great disparity in infectious disease prevalence between developed and developing nations, the WHO divides the world into demographically developed nations (including the United States, Europe, Japan, Australia, and nations of the former Soviet Union) and demographically developing nations (including India, China, other Asian nations, Pacific island nations, Africa, Latin America, and the Middle East) [3]. The WHO lists leading causes of death worldwide by disease categories. The disease can be caused by multiple diverse organisms or co-infections of different organisms, as is the case for acute respiratory disease, or by specific organisms, such as acquired immune deficiency syndrome (AIDS) or tuberculosis. In the former case, we identified the most prominent organisms responsible for the disease for display in Additional File 1.

The CDC-notifiable-disease list represents those diseases that offer the greatest threat to public health in the United States. Additional File 2 [see Additional File 2] lists the agents on this list. There are no set criteria for inclusion on the notifiable disease list; rather, the list is created by the CDC in cooperation with state health departments. As diseases occur less frequently and new diseases emerge, the notifiable disease list changes. An up-to-date version of the list can be found online on the CDC website [74]. The online list provides links to case definitions of each disease, including the etiological agent(s) responsible. In cases where the etiological agent was not listed or was unspecific (i.e. *Brucella *species), further research was done to determine an etiological agent and literature used is cited in legend for Additional File 2.

The most common causative agents of food- and water-borne illness were taken from a publication by Tauxe [75] and are listed in Additional File 3 [see Additional File 3]. Hubálek [20] published a list that constitutes a representative subset of emerging infectious diseases, which was used as the basis for Additional File 4 [see Additional File 4]. The complete list of all species classified as emerging is substantial [18,76]. The number of pathogens listed in the emerging disease table at  is significantly larger than that found in Additional File 4.

Additional File 5 [see Additional File 5]was taken from The *NIAID Strategic Plan For Biodefense Research*, which lists agents with potential for "high morbidity and mortality; potential for person-to-person transmission, directly or by vector; low infective dose and high infectivity by aerosol, with commensurate ability to cause large outbreaks; ability to contaminate food and water supplies; lack of a specific diagnostic test and/or effective treatment; lack of a safe and effective vaccine; potential to cause anxiety in the public and in health care workers; and the potential to be weaponized [77]."

The properties that make organisms amenable for use as biological weapons have been discussed extensively [31,32]. For the purposes of the database, we have categorized microorganisms or toxins as *validated bioweapons *if they have been documented as used, or prepared for use, as biological weapons [78]. *Potential bioweapons *are defined as agents that have some properties that are considered useful as bioweapons or agents for terrorism, or that have been the subject of serious bioweapons research and development [31,39]. Greenwood *et al. *used a hierarchical ranking of 50 infectious agents and toxins of biological origin [31] to classify agents as potentially useful biological weapon*s*. Greenwood *et al.*'s 23 highest scoring agents overlapped substantially with our list of *validated bioweapons *and these are shown in Additional File 6 [see Additional File 6].

The agents listed in Additional File 6 and additional agents listed in Additional File 7 [see Additional File 7] could be used in commission of a biocrime. Biocrimes are similar to traditional crimes that harm specific individuals except that the weapon is biological in nature. A number of biological agents have been used to commit biocrimes [52,79-96], and the potential list of agents that can be used is enormous. Indeed, it is impossible to anticipate the next organism that will be used for illicit purposes. Documented cases and accessibility were the criteria for choice of agents in Additional File 7.

The high potential for bioengineering category [see Additional File 8] includes both organisms that have been actually modified in biological weapons research programs in the past, and organisms with properties that make them amenable to future engineering. Placement in the latter category was based upon published reports of bioengineering for peaceful purposes [53-58] and the author's (DJE) analysis. The agents on the HHS list are shown in Additional File 9. HHS regulates the possession of biological agents and toxins that have the potential to pose a severe threat to public health and safety. HHS's Select Agent Program [97] regularly reviews and updates the list of select agents and critical human pathogens [98].

The USDA is required by federal law to protect animal and plant health. High Consequence Livestock Pathogens and Toxins are agents that the USDA considers to have the potential to pose a severe threat to animal or plant health, or to animal or plant products [99]. These organisms are listed in Additional File 10 [see Additional File 10]. The pathogens on the HHS [see Additional File 9] and USDA lists include agents that span all domains of life, including 20 bacteria, 48 viruses, four fungi, two protists, one prion, and 11 toxins. A significant number of these agents appear on both the HHS and USDA lists.

## Authors' contributions

DJE conceived of the study, organized the structure and contributed to the research and writing, and was overall responsible for the development of the research and the manuscript. RS, VS, TAH, KH, JAM, and JRW contributed to the literature analysis, organization of written sections, and writing of the manuscript. PW conducted extensive research, analysis, and constructed and linked the tables. BB contributed the sections on biocrimes and bioterrorism. CB-O contributed the viral taxonomy components of the manuscript. CM contributed the phylogenetic organization sections, plant pathogen research, and generated the figures.

## Supplementary Material

Additional File 1 ***Globally important human pathogens***. Pathogens are indicated on the phylogenetic charts when they cause at least 0.3 or more deaths per year per hundred thousand population in either developed or developing nations according to WHO estimates for the year 2,000. Medically important organism cause fewer deaths than this threshold, but are considered important agents in western medicine [3,100-104]. Click here for file

Additional File 2 ***CDC notifiable agents***. Literature used in conversion of disease names to pathogen responsible included: [105-116]. Click here for file

Additional File 3 ***Food-borne pathogens***. Pathogens are represented on the phylogenetic charts if they cause more than 1000 cases of food-borne illness per year, estimated for 1997. Literature used in population of this table included: [16,113,114,117-119]. Click here for file

Additional File 4 ***Organisms responsible for emerging infectious diseases***. Literature used in conversion of disease names to pathogen responsible and in population of the table included: [9,18,20,76,106-108,112,116,120-138]. Click here for file

Additional File 5 ***NIAID priority pathogens***. Literature used in population of the table included: [108,114,117,139,140]. Click here for file

Additional File 6 ***Validated and potential biological weapons***. Literature used in population of the table included: [31,39,114,141-144]. Click here for file

Additional File 7 ***Validated and potential biocrimes weapons***. Literature used in population of the table included: [52,79-96]. Click here for file

Additional File 8 ***Agents highly amenable to biological engineering***. Literature used in population of this table included: [53-58]. Click here for file

Additional File 9 ***HHS Select Agents***. Literature used in population of the table included: [145-147]. Click here for file

Additional File 10 ***USDA high consequence pathogens [PDF]***. Literature used in population of the table included: [146,148]. Click here for file
