# Supplementary material for: The Microbial Rosetta Stone Database: A compilation of global and emerging infectious microorganisms and bioterrorist threat agents
Source: BMC Microbiol. 2005 Apr 25;5:19. doi: 10.1186/1471-2180-5-19 (PMC1127111; doi:10.1186/1471-2180-5-19)
Supplement: Additional File 1 — Globally important human pathogens. Pathogens are indicated on the phylogenetic charts when they cause at least 0.3 or more deaths per year per hundred thousand population in either developed or developing nations according to WHO estimates for the year 2,000. Medically important organism cause fewer deaths than this threshold, but are considered important agents in western medicine [3,100-104]. [file 1471-2180-5-19-S1.pdf]

Additional File 1. Globally Important Human Pathogens

Infectious causes of death per year per 100,000 population (estimated for the year 2,000)

Total	Developed nations	Developing nations	Disease	Primary Organisms
98.6	35.3	63.3	Respiratory infections	Haemophilus influenzae, Staphylococcus aureus, Mycoplasma pneumoniae, Chlamydia trachomatis, Bordetella pertussis,Streptococcus pneumoniae, Chlamydophila pneumoniae<br>Human adenovirus<br>Human respiratory syncytial virus, Human parainfluenza viruses 1 and 3, Influenza A Virus, Influenza B Virus
45.6	0.5	45.1	Diarrhoeal diseases	Vibrio cholerae, Shigella dysenteriae, Salmonella typhi, Escherichia coli, Campylobacter, Clostridium difficile, Listeria monocytogenes, Salmonella enteritidis, Norwalk virus, rotavirus<br>Cryptosporidium parvum, Cyclospora cayetanensis, Giardia intestinalis, Encephalitozoon intestinalis, Entamoeba histolytica<br>Norwalk Virus<br>Rotavirus
44.2	2.7	41.5	Tuberculosis	Mycobacterium tuberculosis
33.5	9.1	24.4	HIV	Human immunodeficiency virus 1, Human immunodeficiency virus 2
17.1	-	17.1	Measles	Measles Virus
14.9	-	14.9	Malaria [100]	Plasmodium falciparum, Plasmodium vivax
7.7	-	7.7	Tetanus	Clostridium tetani
5.5	-	5.5	Pertussis	Bordetella pertussis
3.3	-	3.3	Syphilis	Treponema pallidum
3.0	0.8	2.3	Bacterial meningitis [101]	Streptococcus pneumoniae, Neisseria meningitidis, Haemophilus influenzae
1.8	0.3	1.4	Hepatitis B & C	Hepatitus C Virus<br>Hepatitis B Virus
0.6	-	0.6	Trypanosomiasis [102]	Trypanosoma brucei gambiense, Trypanosoma brucei rhodesiense
0.5	-	0.5	Leishmaniasis [103]	Leishmania tropica, Leishmania donovani, Leishmania mexicana venezuelensis, Leishmania garnhami, Leishmania pifanoi, Leishmania braziliensis, Leishmania peruviana, Leishmania colombiensis, Leishmania lainsoni, Leishmaina shawi, Leishmania naiffi, Leishmania guyanensis, Leishmania panamensis, Leishmania chagasi
0.4	-	0.4	Otitis media [104]	Haemophilus influenzae, Streptococcus pneumoniae, Streptococcus pyogenes, Moraxella catarrhalis
0.4	-	0.4	Poliomyelitis	Poliovirus
0.3	-	0.3	Chagas disease [102]	Trypanosoma cruzi
