# Supplementary material for: The Microbial Rosetta Stone Database: A compilation of global and emerging infectious microorganisms and bioterrorist threat agents
Source: BMC Microbiol. 2005 Apr 25;5:19. doi: 10.1186/1471-2180-5-19 (PMC1127111; doi:10.1186/1471-2180-5-19)
Supplement: Additional File 2 — CDC notifiable agents. Literature used in conversion of disease names to pathogen responsible included: [105-116]. [file 1471-2180-5-19-S2.pdf]

Additional File 2A. CDC Notifiable Agents (Cellular)

Phylogeny		*NCBI Name*	Threat List Name or Synonym		Accession			
Fungi	Ascomycota	*Coccidioides immitis*	Coccidioidomycosis					
Eukaryota	Alveolata	*Cryptosporidium parvum*	Cryptosporidiosis				http://www.parvum.mic.vcu.edu/	
		Cyclospora cayetanensis	Cyclosporiasis					
		Plasmodium vivax	Malaria					
		Plasmodium falciparum	Malaria		NC_004325			
	Diplomonadida	*Giardia intestinalis*	Giardiasis / Giardia lamblia					
	Metazoa	*Trichinella britovi*	Trichinosis [105]					
		Trichinella nativa	Trichinosis [105]					
		Trichinella nelsoni	Trichinosis [105]					
		Trichinella pseudospiralis	Trichinosis [105]					
		Trichinella spiralis	Trichinosis [105]					
Bacteria	Alphaproteobacteria	*Anaplasma phagocytophilum*	Human granulocytic ehrlichiosis [106,107]		NC_004351*			
		Brucella melitensis	Brucellosis [108]		NC_003317	NC_003318		
		Brucella melitensis biovar Abortus	Brucellosis [108]					
		Brucella melitensis biovar Suis	Brucellosis [108]		NC_004310	NC_004311		
		Brucella melitensis biovar Canis	Brucellosis [108]					
		Ehrlichia chaffeensis	Human monocytic ehrlichiosis [109]		NC_004127*			
		Ehrlichia canis	Ehrlichiosis [109]					
		Ehrlichia ewingii	Ehrlichiosis [110]					
		Neorickettsia sennetsu	Ehrlichiosis [110]					
		Rickettsia rickettsii	Rocky Mountain Spotted Fever [111]		NZ_AADJ01000001			
	Betaproteobacteria	*Bordetella pertussis*	Pertussis		NC_002929			
		Neisseria gonorrhoeae	Gonorrhea		NC_002946			
		Neisseria meningitidis	Meningococcal disease [112]		NC_003112	NC_003116		
	Gammaproteobacteria	*Coxiella burnetii*	Q Fever		NC_002971			
		Escherichia coli	Enterohemorrhagic E. coli, Shiga-toxin positive non-0157					
		Escherichia coli O157:H7	Enterohemorrhagic E. coli, 0157:H7	Hemolytic uremic syndrome, post-diarrheal	NC_002695	NC_002655		
		Francisella tularensis	Tularemia					
		Haemophilus ducreyi	Chancroid		NC_002940			
		Haemophilus influenzae	Haemophilus influenzae, invasive disease		NC_000907			
		Legionella pneumophila	Legionellosis		NC_002942*		http://genome3.cpmc.columbia.edu/~legion/	
		Salmonella enteritidis	Salmonellosis [113]		NC_002962			
		Salmonella typhi	Typhoid fever		NC_003198*		http://www.sanger.ac.uk/Projects/S_typhi/	
		Salmonella typhimurium	Salmonellosis [113]		NC_003197			
		Shigella boydii	Shigellosis [114]					
		Shigella dysenteriae	Shigellosis [114]	Hemolytic uremic syndrome, post-diarrheal	NC_004510*		http://www.sanger.ac.uk/Projects/Escherichia_Shigella/	
		Shigella flexneri	Shigellosis [114]		NC_004337	NC_004741		
		Shigella sonnei	Shigellosis [114]		NC_004511*		http://www.sanger.ac.uk/Projects/Escherichia_Shigella/	
		Vibrio cholerae O1	Cholera		NC_002505	NC_002506		
		Vibrio cholerae O139	Cholera					
		Yersinia pestis	Plague		NC_003143	NC_004088		
	Firmicutes	*Bacillus anthracis*	Anthrax		NC_003997	NC_003995*		
		Clostridium botulinum	Botulism		NC_003223*		http://www.sanger.ac.uk/Projects/C_botulinum/	
		Clostridium tetani	Tetanus		NC_004557			
		Listeria monocytogenes	Listeriosis		NC_003210			
		Staphylococcus aureus	Toxic-shock syndrome	Vancomycin intermediate / resistant S. aureus	NC_002745	NC_002758	NC_003923	
		Streptococcus pneumoniae	Streptococcus pneumoniae, invasive	Streptococcus pneumoniae, invasive, children <5	NC_003028	NC_003098		
		Streptococcus pyogenes	Streptococcal disease, invasive, group A [115]	Streptococcal toxic-shock syndrome	NC_002737	NC_003485	NC_004070	NC_004606
	Actinobacteria	*Corynebacterium diphtheriae*	Diphtheria		NC_002935			
		Mycobacterium leprae	Hansen disease (leprosy)		NC_002677			
		Mycobacterium tuberculosis	Tuberculosis		NC_002755	NC_000962		
	Chlamydia	*Chlamydophila psittaci*	Psittacosis					
		Chlamydia trachomatis	Chlamydia trachomatis, genital infections		NC_000117			
	Spirochaetes	*Borrelia burgdorferi*	Lyme disease		NC_001318			
		Treponema pallidum	Syphilis	Syphilis, congenital	NC_000919			

Additional File 2B. CDC Notifiable Agents (Viral)

Phylogeny		NCBI Name	Threat List Name or Synonym			Accession		
DNA Virus	Herpesviridae	*Human herpesvirus 3*	Varicella (morbidity)	Varicella (deaths only)		NC_001348		
	Poxviridae	*Variola major virus*	Smallpox			NC_001611		
- Strand RNA Virus	Bunyaviridae	*Andes virus*	Hantavirus Pulmonary Syndrome [116]			NC_003468	NC_003467	NC_003466
		Araraquara virus	Hantavirus Pulmonary Syndrome [116]					
		Bayou virus	Hantavirus Pulmonary Syndrome [116]					
		Bermejo virus	Hantavirus Pulmonary Syndrome [116]					
		Black Creek Canal virus	Hantavirus Pulmonary Syndrome [116]					
		California encephalitis virus	Encephalitis/meningitis, California serogroup					
		Castelo dos Sonhos virus	Hantavirus Pulmonary Syndrome [116]					
		HU39694 virus	Hantavirus Pulmonary Syndrome [116]					
		Laguna Negra virus	Hantavirus Pulmonary Syndrome [116]					
		Lechiguanas virus	Hantavirus Pulmonary Syndrome [116]					
		New York virus	Hantavirus Pulmonary Syndrome [116]					
		Oran virus	Hantavirus Pulmonary Syndrome [116]					
		Sin Nombre virus	Hantavirus Pulmonary Syndrome [116]			NC_005217	NC_005215	NC_005216
	Paramyxoviridae	*Measles virus*	Measles			NC_001498		
		Mumps virus	Mumps			NC_002200		
	Rhabdoviridae	*Rabies virus*	Rabies, human	Rabies, animal		NC_001542		
+ Strand RNA Virus	Flaviviridae	*Hepatitis C*	Hepatitis C, acute	Hepatitis C Virus Infection		NC_004102		
		Powassan virus	Encephalitis/meningitis, Powassan			NC_003687		
		St. Louis encephalitis virus	Encephalitis/meningitis, St. Louis					
		West Nile virus	Encephalitis/meningitis, West Nile			NC_001563		
		Yellow fever virus	Yellow fever			NC_002031		
	Coronaviridae	*SARS Coronavirus*	Severe Acute Respiratory Syndrome-associated Coronavirus (SARS-CoV) disease			NC_004718		
	Picornaviridae	*Hepatitis A virus*	Hepatitis A, acute			NC_001489		
		Poliovirus	Poliomyelitis, paralytic			NC_002058		
	Togaviridae	*Eastern equine encephalitis virus*	Encephalitis/meningitis, Eastern equine			NC_003899		
		Rubella virus	Rubella	Rubella, congenital syndrome		NC_001545		
		Western equine encephalomyelitis virus	Encephalitis/meningitis, Western equine			NC_003908		
Retroid virus	Hepadnaviridae	*Hepatitis B virus*	Hepatitis B virus, perinatal infection	Hepatitis B, acute	Chronic Hepatitis B	NC_003977		
	Retroviridae	*Human immunodeficiency virus 1*	AIDS	HIV infection, pediatric	HIV infection, adult	NC_001802		
		Human immunodeficiency virus 2	AIDS	HIV infection, pediatric	HIV infection, adult	NC_001722		
