# Supplementary material for: The Microbial Rosetta Stone Database: A compilation of global and emerging infectious microorganisms and bioterrorist threat agents
Source: BMC Microbiol. 2005 Apr 25;5:19. doi: 10.1186/1471-2180-5-19 (PMC1127111; doi:10.1186/1471-2180-5-19)
Supplement: Additional File 3 — Food-borne pathogens. Pathogens are represented on the phylogenetic charts if they cause more than 1000 cases of food-borne illness per year, estimated for 1997. Literature used in population of this table included: [16,113,114,117-119]. [file 1471-2180-5-19-S3.pdf]

Additional File 3. Food- and Waterborne Pathogens

Phylogeny		*NCBI Name*	Threat List Name or Synonym	Accession			
Eukaryota	Alveolata	*Cryptosporidium parvum*				http://www.parvum.mic.vcu.edu/	
		Cyclospora cayetanensis					
		Toxoplasma gondii				http://www.sanger.ac.uk/Projects/T_gondii/	
	Diplomonadida	*Giardia intestinalis*	*Giardia lamblia*				
Bacteria	Epsilonproteobacteria	*Campylobacter jejuni*	Campylobacter	NC_002163			
	Gammaproteobacteria	*Escherichia coli*		NC_004431	NC_000913		
		Escherichia coli O157:H7		NC_002695	NC_002655		
		Salmonella typhimurium	Salmonella (nontyphoid) [113]	NC_003197			
		Salmonella enteritidis	Salmonella (nontyphoid) [113]	NC_002962			
		Shigella boydii	Shigella [114]				
		Shigella dysenteriae	Shigella [114]	NC_004510*	http://www.sanger.ac.uk/Projects/Escherichia_Shigella/		
		Shigella flexneri	Shigella [114]	NC_004337	NC_004741		
		Shigella sonnei	Shigella [114]	NC_004511*	http://www.sanger.ac.uk/Projects/Escherichia_Shigella/		
		Vibrio mimicus	Vibrio (noncholera) [117]				
		Vibrio parahaemolyticus	Vibrio (noncholera) [117]				
		Vibrio vulnificus	Vibrio (noncholera) [117]	NC_004603	NC_004605		
		Yersinia enterocolitica		NC_003222*	http://www.sanger.ac.uk/Projects/Y_enterocolitica/		
	Firmicutes	*Bacillus cereus*					
		Listeria monocytogenes		NC_003210			
		Staphylococcus aureus	Staphylococccal food poisoning [118]	NC_002745	NC_002758	NC_003923	
		Streptococcus pyogenes	Streptococci [119]	NC_002737	NC_003485	NC_004070	NC_004606
		Clostridium perfringens		NC_003366			
+ Strand RNA Virus	*Astroviridae*	*Human astrovirus*	Astrovirus	NC_001943			
	Caliciviridae	*Norwalk virus*	Norwalk-like viruses	NC_001959			
	Picornaviridae	*Hepatitis A virus*		NC_001489			
dsRNA Virus	*Reoviridae*	*Rotavirus*	Rotavirus				
Toxin (protein)	Bacteria, Low G+C gram positive, Bacilli	*Staphylococcal enterotoxin A*	Staphylococccal food poisoning [118]	M18970			
		Staphylococcal enterotoxin B	Staphylococccal food poisoning [118]	M11118			
