# Supplementary material for: The Microbial Rosetta Stone Database: A compilation of global and emerging infectious microorganisms and bioterrorist threat agents
Source: BMC Microbiol. 2005 Apr 25;5:19. doi: 10.1186/1471-2180-5-19 (PMC1127111; doi:10.1186/1471-2180-5-19)
Supplement: Additional File 4 — Organisms responsible for emerging infectious diseases. Literature used in conversion of disease names to pathogen responsible and in population of the table included: [9,18,20,76,106-108,112,116,120-138]. [file 1471-2180-5-19-S4.pdf]

Additional File 4A. Emerging Infectious Diseases (Cellular)

Phylogeny		NCBI Name	Common Name / Disease	Accession			
Fungi	Ascomycota	*Ajellomyces capsulatus*	histoplasmosis [20]				
		Histoplasma capsulatum var. duboisii	histoplasmosis [20]				
		Candida spp.	candidiasis	NC_005967			
		Coccidioides immitis	coccidioidomycosis [20]				
		Fusarium oxysporum	fusariosis [20]				
		Fusarium solani	fusariosis [20]				
		Pneumocystis jirovecii	Pneumocystis pneumonia (human genotype) [120]				
	Basidiomycota	*Filobasidiella neoformans*	cryptococcosis [20]				
	Microsporidia		microsporidial infections				
Protists	Acanthamoebidae	*Acanthamoeba castellanii*	amoebic keratitis or chronic granulomatous amoebic meningoencephalitis [20]				
		Acanthamoeba polyphaga	amoebic keratitis or chronic granulomatous amoebic meningoencephalitis [20]				
	Alveolata	*Cryptosporidium parvum*	cryptosporidiosis (human genotype), cryptosporidiosis (calf genotype 2) [121]			http://www.parvum.mic.vcu.edu/	
		Cryptosporidium hominis	cryptosporidiosis (human genotype) [121]				
		Plasmodium vivax	malaria				
		Plasmodium falciparum	malaria	NC_004325			
	Diplomonadida	*Giardia intestinalis*	giardiasis (human genotype) [122]				
	Heterolobosea	*Naegleria fowleri*	primary amebic meningoencephalitis [20]				
Bacteria	Actinobacteria	*Corynebacterium diphtheriae*	diphtheria	NC_002935			
		Corynebacterium xerosis	corynebacterial endocarditis [20]				
		Corynebacterium amycolatum	corynebacterial endocarditis [20]				
		Mycobacterium tuberculosis	tuberculosis (multidrug-resistant strains)	NC_002755	NC_000962		
		Mycobacterium avium	avian tuberculosis	NC_002944			
		Mycobacterium bovis	bovine tuberculosis [123]	NC_002945			
		Mycobacterium ulcerans	Buruli ulcer disease				
		Mycobacterium kansasii	mycobacterial diseases other than tuberculosis [20]				
		Mycobacterium xenopi	mycobacterial diseases other than tuberculosis [20]				
		Mycobacterium marinum	mycobacterial diseases other than tuberculosis [20]	NC_004506*			
		Mycobacterium haemophilum	mycobacterial diseases other than tuberculosis [20]				
		Mycobacterium fortuitum	mycobacterial diseases other than tuberculosis [20]				
		Mycobacterium scrofulaceum	mycobacterial diseases other than tuberculosis [20]				
		Mycobacterium abscessus	mycobacterial diseases other than tuberculosis [20]				
		Rhodococcus equi	rhodococcosis [20]				
	Alphaproteobacteria	*Anaplasma phagocytophilum*	human granulocytic ehrlichiosis [106, 107]	NC_004351*			
		Bartonella quintana	trench fever [124]	NC_005955			
		Bartonella henselae	cat-scratch fever [124]	NC_005956			
		Brucella melitensis	brucellosis [108]	NC_003317	NC_003318		
		Brucella melitensis biovar Abortus	brucellosis [108]				
		Brucella melitensis biovar Suis	brucellosis [108]	NC_004310	NC_004311		
		Rickettsia conorii	African tick typhus [125]	NC_003103			
		Rickettsia typhi	Murine typhus [126, 127]				
	Bacteroidetes	*Chryseobacterium meningosepticum*	atypical bacterial meningitis and sepsis [20]				
	Betaproteobacteria	*Bordetella pertussis*	whooping cough [128]	NC_002929			
		Burkholderia cepacia	bacterial infections associated with cystic fibrosis [20]	NZ_AAEH00000000*		NZ_AAEI00000000*	
		Burkholderia pseudomallei	melioidosis [20]	NC_002930*	http://www.sanger.ac.uk/Projects/B_pseudomallei/		
		Neisseria meningitidis	meningococcal disease [112]	NC_003112	NC_003116		
		Ralstonia spp.	bacterial infections associated with cystic fibrosis [20]				
	Chlamydia	*Chlamydia trachomatis*	chlamydial pneumonia and cardiovascular disease [129]	NC_000117			
		Chlamydophila psittaci	chlamydial pneumonia and cardiovascular disease [129]				
		Chlamydophila pneumoniae	chlamydial pneumonia and cardiovascular disease [129]	NC_005043	NC_002491	NC_002179	NC_000922
		Parachlamydia acanthamoebae	Chlamydia-like pneumonia [20]				
	Epsilonproteobacteria	*Campylobacter jejuni*	campylobacterosis [9]	NC_002163			
		Helicobacter pylori	peptic ulcer disease [130]	NC_000915	NC_000921		
	Firmicutes	*Bacillus cereus*	food poisoning [20]				
		Clostridium difficile	intestinal clostridiosis [20]	NC_002933*			
		Clostridium perfringens	intestinal clostridiosis [20]	NC_003366			
		Enterococcus faecium	vancomycin-resistant enterococcal disease [131, 132]	NZ_AAAK00000000*			
	Gammaproteobacteria	*Acinetobacter calcoaceticus*	acinetobacter bacteremia [20]				
		Acinetobacter baumanii	acinetobacter bacteremia [20]				
		Acinetobacter radioresistans	acinetobacter bacteremia [20]				
		Escherichia coli O157:H7	hemorrhagic colitis, hemolytic uremic syndrome [133]	NC_002695	NC_002655		
		Francisella tularensis	tularemia				
		Haemophilus influenzae	Haemophilus infections (including Brazilian purpuric fever) [134, 135]	NC_000907			
		Haemophilus aegyptius	Haemophilus infections (including Brazilian purpuric fever) [136]				
		Klebsiella pneumoniae	nosocomial Klebsiella pneumoniae	NC_002941			
		Legionella pneumophila	legionellosis and Pontiac fever [20]	NC_002942*	http://genome3.cpmc.columbia.edu/~legion/		
		Tatlockia micdadei	legionellosis and Pontiac fever [20]				
		Pseudomonas aeruginosa	Pseudomonas aeruginosa bacteremia (including antibiotic-resistant strains)				
		Salmonella enteriditis	salmonellosis [137]				
		Salmonella typhimurium	salmonellosis [137]				
		Vibrio parahaemolyticus	vibrio gastroenteritis or dermatitis [20]	NC_004603	NC_004605		
		Vibrio vulnificus	vibrio gastroenteritis or dermatitis [20]	NC_004459	NC_004460	NC_005139	NC_005140
	Spirochaetes	*Borrelia burgdorferi*	Lyme disease [138]	NC_001318			

Additional File 4B. Emerging Infectious Diseases (Viral)

Phylogeny		*Species Name*	*Common Name / Disease*	*Accession*		
dsDNA	*Poxviridae*	Monkeypox Virus	human monkeypox	NC_003310		
ssRNA -	*Arenaviridae*	Guanarito Virus	Venezuelan hemorrhagic fever	NC_005077	NC_005082	
		Sabia Virus	Brazilian hemorrhagic fever			
		Lassa Virus	Lassa hemorrhagic fever	NC_004296	NC_004297	
	Bunyaviridae	Andes Virus	hantavirus pulmonary syndrome [116]			
		Araraquara Virus	hantavirus pulmonary syndrome [116]			
		Bayou Virus	hantavirus pulmonary syndrome [116]			
		Bermejo Virus	hantavirus pulmonary syndrome [116]			
		Black Creek Canal Virus	hantavirus pulmonary syndrome [116]			
		Castelo dos Sonhos Virus	hantavirus pulmonary syndrome [116]			
		Dobrava Virus	hemorrhagic fever with renal syndrome [116]			
		Hantaan Virus	hemorrhagic fever with renal syndrome [116]	NC_005222	NC_005219	NC_005218
		Hu39694 Virus	hantavirus pulmonary syndrome [116]			
		Laguna Negra Virus	hantavirus pulmonary syndrome [116]			
		Lechiguanas Virus	hantavirus pulmonary syndrome [116]			
		New York Virus	hantavirus pulmonary syndrome [116]			
		Oran Virus	hantavirus pulmonary syndrome [116]			
		Puumala Virus	hemorrhagic fever with renal syndrome [116]			
		Seoul Virus	hemorrhagic fever with renal syndrome [116]			
		Sin Nombre Virus	hantavirus pulmonary syndrome [116]	NC_005217	NC_005215	NC_005216
		Crimean-Congo hemorrhagic fever virus	Crimean-Congo hemorrhagic fever	NC_005301	NC_005300	NC_005302
		Rift Valley Fever Virus	Rift Valley fever	NC_002043	NC_002044	NC_002045
	Filoviridae	Ivory Coast ebolavirus	Ebola hemorrhagic fever			
		Reston ebolavirus	Ebola hemorrhagic fever	NC_004161		
		Sudan ebolavirus	Ebola hemorrhagic fever			
		Zaire ebolavirus	Ebola hemorrhagic fever	NC_002549		
		Lake Victoria marburgvirus	Marburg hemorrhagic fever	NC_001608		
	Paramyxoviridae	Hendra Virus	Hendra hemorrhagic bronchopneumonia	NC_001906		
		Nipah Virus	Nipah hemorrhagic bronchopneumonia	NC_002728		
		Measles Virus	Measles	NC_001498		
ssRNA +	*Flaviviridae*	Japanese encephalitis virus	Japanese encephalitis	NC_001437		
		West Nile Virus	West Nile fever	NC_001563		
		Dengue Virus	dengue/dengue hemorrhagic fever	NC_001474		
	Picornaviridae	Coxsackievirus A7	Acute flaccid paralysis			
		Coxsackievirus A9	Acute flaccid paralysis			
		Human enterovirus 71	Acute flaccid paralysis			
	Togaviridae	Chikungunya Virus	Chikungunya fever	NC_004162		
		O'nyong-nyong Virus	O'nyong nyong fever	NC_001512		
		Ross River Virus	Ross River epidemic polyarthritis	NC_001544		
Retroid	*Retroviridae*	Human immunodeficiency Virus 1	AIDS	NC_001802		
		Human immunodeficiency Virus 2	AIDS	NC_001722		
		Human T-lymphotropic virus 1				
		Human T-lymphotropic virus 2				
