# Supplementary material for: The Microbial Rosetta Stone Database: A compilation of global and emerging infectious microorganisms and bioterrorist threat agents
Source: BMC Microbiol. 2005 Apr 25;5:19. doi: 10.1186/1471-2180-5-19 (PMC1127111; doi:10.1186/1471-2180-5-19)
Supplement: Additional File 5 — NIAID priority pathogens. Literature used in population of the table included: [108,114,117,139,140]. [file 1471-2180-5-19-S5.pdf]

Additional File 5A. NIAID Priority Pathogens Category A

Phylogeny		*NCBI Name*	*Agency Name or Synonym*	*Accession*				
Bacteria	Gammaproteobacteria	*Francisella tularensis*						
		Yersinia pestis		NC_003143	NC_004088			
	Firmicutes	*Bacillus anthracis*		NC_003997	NC_003995*	NC_004352*	NC_002925*	NC_004126*
		Clostridium botulinum		NC_003223*		http://www.sanger.ac.uk/Projects/C_botulinum/		
DNA Virus	*Poxviridae*	*Variola major virus*		NC_001611				
- Strand RNA Virus	*Arenaviridae*	*Guanarito virus*		NC_005077	NC_005082			
		Junin virus		NC_005080	NC_005081			
		Lassa virus		NC_004296	NC_004297			
		Lymphocytic choriomeningitis virus	LCM	NC_004291	NC_004294			
		Machupo virus		NC_005078	NC_005079			
	Bunyaviridae	*Andes virus*	Hantaviruses	NC_003468	NC_003467	NC_003466		
		Bayou virus	Hantaviruses					
		Hantaan virus	Hantaviruses	NC_005222	NC_005219	NC_005218		
		Rift Valley fever virus		NC_002043	NC_002044	NC_002045		
		Sin Nombre virus	Hantaviruses	NC_005217	NC_005215	NC_005216		
	Filoviridae	*Ivory Coast ebolavirus*						
		Lake Victoria marburgvirus		NC_001608				
		Reston ebolavirus		NC_004161				
		Sudan ebolavirus						
		Zaire ebolavirus		NC_002549				
+ Strand RNA Virus	*Flaviviridae*	*Dengue virus*						
		Dengue virus type 1						
		Dengue virus type 2		NC_001474				
		Dengue virus type 3						
		Dengue virus type 4						

Additional File 5B. NIAID Priority Pathogens Category B

Phylogeny		NCBI Name	Agency Name or Synonym	Accession			
Fungi	Microsporidia	*Encephalitozoon cuniculi*	Microsporidia [140]	NC_003242			
		Encephalitozoon hellem	Microsporidia [140]				
		Encephalitozoon intestinalis	Microsporidia [140]				
		Enterocytozoon bieneusi	Microsporidia [140]				
Eukaryota	Alveolata	*Cryptosporidium parvum*				http://www.parvum.mic.vcu.edu/	
		Cyclospora cayetanensis					
		Toxoplasma gondii				http://www.sanger.ac.uk/Projects/T_gondii/	
	Diplomonadida	*Giardia intestinalis*					
	Entamoebidae	*Entamoeba histolytica*				http://www.sanger.ac.uk/Projects/E_histolytica/	
Bacteria	Alphaproteobacteria	*Brucella melitensis*	Brucella species [108]	NC_003317	NC_003318		
		Brucella melitensis biovar Abortus	Brucella species [108]				
		Brucella melitensis biovar Canis	Brucella species [108]				
		Brucella melitensis biovar Suis	Brucella species [108]	NC_004310	NC_004311		
		Rickettsia prowazekii	Typhus fever	NC_000963			
	Betaproteobacteria	*Burkholderia mallei*		NC_002970*			
		Burkholderia pseudomallei		NC_002930*		http://www.sanger.ac.uk/Projects/B_pseudomallei/	
	Epsilonproteobacteria	*Campylobacter jejuni*		NC_002163			
	Gammaproteobacteria	*Coxiella burnetii*		NC_002971			
		Escherichia coli		NC_004431	NC_000913		
		Escherichia coli O157:H7		NC_002695	NC_002655		
		Salmonella typhi	Salmonella	NC_003198*		http://www.sanger.ac.uk/Projects/S_typhi/	
		Shigella boydii	Shigella species [114]				
		Shigella dysenteriae	Shigella species [114]	NC_004510*		http://www.sanger.ac.uk/Projects/Escherichia_Shigella/	
		Shigella flexneri	Shigella species [114]	NC_004337	NC_004741		
		Shigella sonnei	Shigella species [114]	NC_004511*		http://www.sanger.ac.uk/Projects/Escherichia_Shigella/	
		Vibrio cholerae	Pathogenic Vibrios [117]	NC_002505	NC_002506		
		Vibrio cholerae O139	Pathogenic Vibrios [117]				
		Vibrio mimicus	Pathogenic Vibrios [117]				
		Vibrio parahaemolyticus	Pathogenic Vibrios [117]	NC_004603	NC_004605		
		Vibrio vulnificus	Pathogenic Vibrios [117]	NC_004459	NC_004460	NC_005139	NC_005140
		Yersinia enterocolitica		NC_003222*		http://www.sanger.ac.uk/Projects/Y_enterocolitica/	
	Firmicutes	*Listeria monocytogenes*		NC_003210			
- Strand RNA Virus	*Bunyaviridae*	*California encephalitis virus*					
		La Crosse virus		NC_004108	NC_004109	NC_004110	
+ Strand RNA Virus	*Caliciviridae*	*Norwalk virus*	Caliciviruses	NC_001959			
	Flaviviridae	*Japanese encephalitis virus*		NC_001437			
		Kyasanur forest disease virus					
		West Nile virus		NC_001563			
	Picornaviridae	*Hepatitis A virus*		NC_001489			
	Togaviridae	*Eastern equine encephalitis virus*	EEE	NC_003899			
		Venezuelan equine encephalitis virus	VEE	NC_001449			
		Western equine encephalomyelitis virus	WEE	NC_003908			
Toxin (protein)	Bacteria, Low G+C gram positive, Clostridia	*Clostridium perfringens epsilon toxin*		M95206	M80837		
	Bacteria, Low G+C gram positive, Bacilli	*Staphylococcal enterotoxin B*		M11118			
	Plant, embryophyta	*Ricin*					

Additional File 5C. NIAID Priority Pathogens Category C

Phylogeny		NCBI Name	Agency Name or Synonym	Accession		
Bacteria	Alphaproteobacteria	*Rickettsia conorii*	Other Rickettsias [140]	NC_003103		
		Rickettsia rickettsii	Other Rickettsias [140]	NZ_AADJ01000001		
		Rickettsia typhi	Other Rickettsias [140]			
	Actinobacteria	*Mycobacterium tuberculosis*		NC_002755	NC_000962	
- Strand RNA Virus	*Bunyaviridae*	*Crimean-Congo hemorrhagic fever virus*		NC_005301	NC_005300	NC_005302
	Orthomyxoviridae	*Influenza A virus*		NC_004905	NC_004518	NC_002016
		Influenza B virus		NC_004791	NC_004784	NC_002204
		Influenza C virus				
	Rhabdoviridae	*Rabies virus*		NC_001542		
+ Strand RNA Virus	*Flaviviridae*	*Tick-borne encephalitis virus*		NC_001672		
		Yellow fever virus		NC_002031		
	Coronaviridae	*SARS coronavirus*		NC_004718		
