# Supplementary material for: The Microbial Rosetta Stone Database: A compilation of global and emerging infectious microorganisms and bioterrorist threat agents
Source: BMC Microbiol. 2005 Apr 25;5:19. doi: 10.1186/1471-2180-5-19 (PMC1127111; doi:10.1186/1471-2180-5-19)
Supplement: Additional File 6 — Validated and potential biological weapons. Literature used in population of the table included: [31,39,114,141-144]. [file 1471-2180-5-19-S6.pdf]

Additional File 6A. Validated and Potential Biological Weapons (Cellular)

Phylogeny		NCBI Name	Threat List Name or Synonym	Validated / Potential	Accession
Fungi	Ascomycota	*Ajellomyces capsulatus*	Histoplasma capsulatum / histoplasmosis	Potential	
		Coccidioides immitis	Coccidioiomycosis	Potential	
	Basidiomycota	*Filobasidiella neoformans*	Cryptococcus neoformans / cryptococcosis	Potential	http://rcweb.bcgsc.bc.ca/cgi-bin/cryptococcus/cn.pl
		Ustilago tritici		Validated	
Bacteria	Alphaproteobacteria	*Brucella melitensis*	Brucellosis / Micrococcus melitensis / Streptococcus miltensis / Brucella meltensis	Validated	NC_003317 NC_003318
		Brucella melitensis biovar Abortus	Brucellosis / Brucella abortus/ Bacterium abortus	Validated	
		Brucella melitensis biovar Suis	Brucellosis / Brucella suis	Validated	NC_004310 NC_004311
		Orientia tsutsugamushi	Rickettsia tsutsugamushi / Rickettsia akamushi / Rickettsia orientalis / Theileria tsutsugamushi	Potential	
		Rickettsia prowazekii	Typhus	Validated	NC_000963
		Rickettsia rickettsii	Rocky Mountain Spotted Fever	Potential	
		Rickettsia typhi	Rickettsia mooseri / murine typhus	Potential	
	Betaproteobacteria	*Burkholderia mallei*	Glanders	Validated	NC_002970*
		Burkholderia pseudomallei	Meliodosis / Pseudomonas pseudomallei	Validated	NC_002930* http://www.sanger.ac.uk/Projects/B_pseudomallei/
	Gammaproteobacteria	*Coxiella burnetii*	Q fever	Validated	NC_002971
		Francisella tularensis	Tularemia / Francisella novicida	Validated	
		Legionella pneumophila	legionellosis	Potential	NC_002942* http://genome3.cpmc.columbia.edu/~legion/
		Salmonella typhi	typhoid / Bacillus typhi	Potential	NC_003198* http://www.sanger.ac.uk/Projects/S_typhi/
		Shigella dysenteriae	Shigella	Validated	NC_004510* http://www.sanger.ac.uk/Projects/Escherichia_Shigella/
		Vibrio cholerae	Cholera	Validated	NC_002505 NC_002506
		Vibrio cholerae O139		Validated	
		Yersinia pestis	Plague	Validated	NC_003143 NC_004088
	Firmicutes	*Bacillus anthracis*	Anthrax	Validated	NC_003997 NC_003995* NC_004352* NC_002925* NC_002925*
		Clostridium perfringens		Validated	NC_003366
	Actinobacteria	*Nocardia asteroides*	nocardiosis / Actinomyces asteroides / Actinomyces eppingeri	Potential	
	Chlamydia	*Chlamydophila psittaci*	Chlamydia psittaci / psittacosis	Potential	
Toxin (protein)	Bacteria, Low G+C gram positive, Clostridia	*Botulinum toxin*	Botulinal toxins	Validated	AF488749 AB088207 AB082519
		Clostridium perfringens epsilon toxin		Validated	M95206 M80837
	Bacteria, Low G+C gram positive, Bacilli	*Staphylococcal enterotoxin B*		Validated	M11118
	Bacteria, gammaproteobactera	*Shigatoxin*		Potential	AB035142 AB035143 AF461169 AF461170 AF461170
	Animal, mollusca, gastropoda	*Conotoxins*		Potential	
	Plant, embryophyta	*Ricin*		Validated	
Toxins (small molecule)	Bacteria, gammaproteobacteria	*Tetrodotoxin*		Potential	
	Protists, alveolata	*Saxitoxin*		Potential	
	Fungi, ascomycota	*Aflatoxin*	mycotoxins	Validated	
		Diacetoxyscirpenol	mycotoxins	Validated	
		T-2 toxin	mycotoxins	Validated	
	Animal, chordata, amphibia	*Batrachotoxin*		Potential	

Additional File 6B. Validated and Potential Biological Weapons (Viral)

Phylogeny		NCBI Name	Threat List Name or Synonym	Validated / Potential	Accession		
DNA Virus	Poxviridae	*Monkeypox virus*		Potential	NC_003310		
		Variola major virus	Smallpox	Validated	NC_001611		
- Strand RNA Virus	Arenaviridae	*Junin virus*	Junin arenavirus	Potential	NC_005080	NC_005081	
		Lassa virus		Potential	NC_004296	NC_004297	
		Lymphocytic choriomeningitis virus	LCMV	Potential	NC_004291	NC_004294	
		Machupo virus		Potential	NC_005078	NC_005079	
	Bunyaviridae	*Crimean-Congo hemorrhagic fever virus*	CCHFV	Potential	NC_005301	NC_005300	NC_005302
		Hantaan virus	Korean hemorrhagic fever virus	Potential	NC_005222	NC_005219	NC_005218
		Rift Valley fever virus	RVFV / Lunyo virus	Potential	NC_002043	NC_002044	NC_002045
		Seoul virus	epidemic hemorrhagic fever virus	Potential	NC_005238	NC_005237	NC_005236
	Filoviridae	*Ivory Coast ebolavirus*	CIEBOV / Cote d'Ivorie Ebola virus	Potential			
		Lake Victoria marburgvirus	Lake Victoria marburgvirus	Potential	NC_001608		
		Reston ebolavirus	REBOV / Ebola virus Reston	Potential	NC_004161		
		Sudan ebolavirus	SEBOV / Ebolavirus Sudan	Potential			
		Zaire ebolavirus	ZEBOV	Potential	NC_002549		
+ Strand RNA Virus	Flaviviridae	*Dengue virus*		Potential			
		Dengue virus type 1	DEN-1	Potential			
		Dengue virus type 2	DEN-2	Potential	NC_001474		
		Dengue virus type 3		Potential			
		Dengue virus type 4	DEN4	Potential			
		Japanese encephalitis virus	JEV	Potential	NC_001437		
		Kyasanur forest disease virus		Potential			
		Omsk hemorrhagic fever virus	OHF	Potential	NC_005062		
		Russian Spring-Summer encephalitis virus	RSSE	Potential			
		St. Louis encephalitis virus	SLEV	Potential			
		Yellow fever virus	YFV / Flavivirus febricis	Potential	NC_002031		
	Togaviridae	*Chikungunya virus*		Potential	NC_004162		
		Eastern equine encephalitis virus	EEE / EEEV / Eastern equine encephalomyelitis virus	Potential	NC_003899		
		O'nyong-nyong virus	ONN	Potential	NC_001512		
		Ross River virus	RRV	Potential	NC_001544		
		Venezuelan equine encephalitis virus	VEE / Venezuelan equine encephalomyelitis virus	Validated	NC_001449		
		Western equine encephalomyelitis virus	Western equine encephalitis virus	Validated	NC_003908		
