# Supplementary material for: The Microbial Rosetta Stone Database: A compilation of global and emerging infectious microorganisms and bioterrorist threat agents
Source: BMC Microbiol. 2005 Apr 25;5:19. doi: 10.1186/1471-2180-5-19 (PMC1127111; doi:10.1186/1471-2180-5-19)
Supplement: Additional File 7 — Validated and potential biocrimes weapons. Literature used in population of the table included: [52,79-96]. [file 1471-2180-5-19-S7.pdf]

Additional File 7. Bioterror and Biocrime Agents

Phylogeny		*NCBI Name*	Threat List Name or Synonym	Accession				
Eukaryota	Chromadorea	*Ascaris suum*	pig roundworm					
	Diplomonadida	*Giardia intestinalis*	*Giardia lamlia / Giardia duodenalis*					
Bacteria	Alphaproteobacteria	*Rickettsia prowazekii*	Typhus	NC_000963				
	Betaproteobacteria	*Burkholderia mallei*	Glanders	NC_002970*				
	Gammaproteobacteria	*Coxiella burnetii*	Q fever	NC_002971				
		Salmonella paratyphi		NC_002963*	http://genome.wustl.edu/projects/bacterial/sparatyphiB/			
		Salmonella typhi	Bacillus typhi	NC_003198*	http://www.sanger.ac.uk/Projects/S_typhi/			
		Salmonella typhimurium		NC_003197	NC_004509*			
		Shigella dysenteriae		NC_004510*	http://www.sanger.ac.uk/Projects/Escherichia_Shigella/			
		Vibrio cholerae	cholera	NC_002505	NC_002506			
		Vibrio cholerae O139						
		Yersinia enterocolitica		NC_003222*	http://www.sanger.ac.uk/Projects/Y_enterocolitica/			
		Yersinia pestis	plague	NC_003143	NC_004088			
	Firmicutes	*Bacillus anthracis*	anthrax	NC_003997	NC_003995*	NC_004352*	NC_002925*	NC_004126*
		Clostridium botulinum		NC_003223*	http://www.sanger.ac.uk/Projects/C_botulinum/			
		Clostridium tetani	Tetanus	NC_004557				
	Actinobacteria	*Corynebacterium diphtheriae*		NC_002935				
		Mycobacterium tuberculosis	Tuberculosis	NC_002755	NC_000962			
+ Strand RNA Virus	*Caliciviridae*	*Rabbit hemorrhagic disease virus*	RHDV	NC_001543				
	Flaviviridae	*Hepatitis C Virus*	HCV	NC_004102				
Retroid virus	*Retroviridae*	*Human immunodeficiency virus 1*	HIV-1	NC_001802				
		Human immunodeficiency virus 2	HIV-2	NC_001722				
Toxin (protein)	Bacteria, Low G+C gram positive, Clostridia	*Botulinum toxin*		AF488749	AB088207	AB082519		
		Clostridium perfringens epsilon toxin		M95206	M80837			
	Bacteria, gammaproteobactera	*Cholera endotoxin*		D30053	D30052	AF390572		
	Bacteria, actinobacteria	*Diphtheria toxin*		A04646	AY141013	AY141014		
	Plant, embryophyta	*African milk bush toxin*						
		Abrin						
		Ricin						
Toxin (small molecule)	Plant, embryophyta	*Nicotine*						
