# Supplementary material for: The Microbial Rosetta Stone Database: A compilation of global and emerging infectious microorganisms and bioterrorist threat agents
Source: BMC Microbiol. 2005 Apr 25;5:19. doi: 10.1186/1471-2180-5-19 (PMC1127111; doi:10.1186/1471-2180-5-19)
Supplement: Additional File 8 — Agents highly amenable to biological engineering. Literature used in population of this table included: [53-58]. [file 1471-2180-5-19-S8.pdf]

Additional File 8. High Potential For Bioengineering

Phylogeny		NCBI Name	Threat List Name or Synonym	Accession		
Bacteria	Alphaproteobacteria	*Brucella melitensis*	*Brucella* spp.	NC_003317	NC_003318	
		Brucella melitensis biovar Abortus	*Brucella* spp.			
		Brucella melitensis biovar Suis	*Brucella* spp.	NC_004310	NC_004311	
		Rickettsia rickettsii		NZ_AADJ01000001		
	Betaproteobacteria	*Burkholderia mallei*		NC_002970*		
		Burkholderia pseudomallei		NC_002930*		http://www.sanger.ac.uk/Projects/B_pseudomallei/
	Gammaproteobacteria	*Coxiella burnetii*		NC_002971		
		Escherichia coli		NC_004431	NC_000913	
		Francisella tularensis				
		Vibrio cholerae		NC_002505	NC_002506	
		Yersinia pseudotuberculosis				
		Yersinia pestis		NC_003143	NC_004088	
	Firmicutes	*Bacillus anthracis*		NC_003997	NC_003995*	
		Staphylococcus aureus		NC_002745	NC_002758	NC_003923
	Spirochaetes	*Borrelia burgdorferi*		NC_001318		
DNA Virus	*Parvoviridae*	*Adeno-associated virus 1*		NC_002077		
		Adeno-associated virus 2		NC_001401		
		Adeno-associated virus 2H				
		Adeno-associated virus 3		NC_001729		
		Adeno-associated virus 3B		NC_001863		
		Adeno-associated virus 4		NC_001829		
		Adeno-associated virus 5				
		Adeno-associated virus 6		NC_001862		
		Adeno-associated virus 7				
		Adeno-associated virus 8				
		Adeno-associated virus 9				
		Adeno-associated virus 10				
		Adeno-associated virus 11				
		Adeno-associated virus 12				
	Poxviridae	*Aracatuba virus*				
		BeAn 58058 virus				
		Buffalopox virus				
		Camelpox virus		NC_003391		
		Cantagalo orthopoxvirus				
		Cowpox virus		NC_003663		
		Dolphin poxvirus 1				
		Ectromelia virus		NC_004105		
		Elephantpox virus				
		Monkeypox virus		NC_003310		
		Rabbitpox virus				
		Skunkpox virus				
		SPAN 232 virus				
		Steller sea lion poxvirus				
		Taterapox virus				
		Vaccinia virus		NC_001559		
		Variola major virus		NC_001611		
		Variola minor virus				
		Volepox virus				
- Strand RNA Virus	*Arenaviridae*	*Junin virus*		NC_005080	NC_005081	
		Lassa virus		NC_004296	NC_004297	
	Bunyaviridae	*California encephalitis virus*				
		Sin Nombre virus		NC_005217	NC_005215	NC_005216
	Filoviridae	*Ivory Coast ebolavirus*				
		Lake Victoria marburgvirus		NC_001608		
		Reston ebolavirus		NC_004161		
		Sudan ebolavirus				
		Zaire ebolavirus		NC_002549		
	Orthomyxoviridae	*Influenza A virus*		NC_004905	NC_004518	NC_002016
		Influenza B virus		NC_004791	NC_004784	NC_002204
		Influenza C virus				
+ Strand RNA Virus	*Coronaviridae*	*SARS Coronavirus*		NC_004718		
	Flaviviridae	*Dengue virus*				
		Dengue virus type 1				
		Dengue virus type 2		NC_001474		
		Dengue virus type 3				
		Dengue virus type 4				
		Japanese encephalitis virus		NC_001437		
		Tick-borne encephalitis virus		NC_001672		
		West Nile virus		NC_001563		
		Yellow fever virus		NC_002031		
	Togaviridae	*Eastern equine encephalitis virus*		NC_003899		
		Venezuelan equine encephalitis virus		NC_001449		
		Western equine encephalomyelitis virus		NC_003908		
