# Supplementary material for: The Microbial Rosetta Stone Database: A compilation of global and emerging infectious microorganisms and bioterrorist threat agents
Source: BMC Microbiol. 2005 Apr 25;5:19. doi: 10.1186/1471-2180-5-19 (PMC1127111; doi:10.1186/1471-2180-5-19)
Supplement: Additional File 9 — HHS Select Agents. Literature used in population of the table included: [145-147]. [file 1471-2180-5-19-S9.pdf]

Additional File 9. HHS Select Agents

Phylogeny		NCBI Name	Threat List Name or Synonym	Accession
Fungi	Ascomycota	*Coccidioides immitis*		
		Coccidioides posadasii		
Bacteria	Alphaproteobacteria	*Brucella melitensis*		NC_003317 NC_003318
		Brucella melitensis biovar Abortus	*Brucella abortus*	
		Brucella melitensis biovar Suis	*Brucella suis*	NC_004310 NC_004311
		Rickettsia prowazekii		NC_000963
		Rickettsia rickettsii		NZ_AADJ01000001
	Betaproteobacteria	*Burkholderia mallei*	*Pseudomonas mallei*	NC_002970*
		Burkholderia pseudomallei	*Pseudomonas pseudomallei*	NC_002930* http://www.sanger.ac.uk/Projects/B_pseudomallei/
	Gammaproteobacteria	*Coxiella burnetii*		NC_002971
		Francisella tularensis		
		Yersinia pestis		NC_003143 NC_004088
	Firmicutes	*Bacillus anthracis*		NC_003997 NC_003995* NC_004352* NC_002925* NC_004126*
		Clostridium botulinum	Botulinum neurotoxin producing species of *Clostridium* [146]	NC_003223* http://www.sanger.ac.uk/Projects/C_botulinum/
		Clostridium baratii	Botulinum neurotoxin producing species of *Clostridium* [146]	
		Clostridium butyricum	Botulinum neurotoxin producing species of *Clostridium* [146]	
DNA Virus	*Herpesviridae*	*Cercopithecine herpesvirus 1*		NC_004812
	Poxviridae	*Monkeypox virus*		NC_003310
		Variola major virus		NC_001611
		Variola minor virus		
- Strand RNA Virus	*Arenaviridae*	*Flexal virus*		
		Guanarito virus		NC_005077 NC_005082
		Junin virus		NC_005080 NC_005081
		Lassa virus		NC_004296 NC_004297
		Machupo virus		NC_005078 NC_005079
		Sabia virus		
	Bunyaviridae	*Crimean-Congo hemorrhagic fever virus*		NC_005301 NC_005300 NC_005302
		Rift Valley fever virus		NC_002043 NC_002044 NC_002045
	Filoviridae	*Ivory Coast ebolavirus*	*Ebola viruses*	
		Lake Victoria marburgvirus		NC_001608
		Reston ebolavirus	*Ebola viruses*	NC_004161
		Sudan ebolavirus	*Ebola viruses*	
		Zaire ebolavirus	*Ebola viruses*	NC_002549
	Paramyxoviridae	*Hendra virus*		NC_001906
		Nipah virus		NC_002728
+ Strand RNA Virus	*Flaviviridae*	*Kumlinge virus*	*Central European tick-borne encephalitis Virus*	
		Kyasanur forest disease virus		
		Omsk hemorrhagic fever virus		NC_005062
		Russian Spring-Summer encephalitis virus		
		Tick-borne encephalitis virus		NC_001672
		Tick-borne encephalitis virus (STRAIN SOFJIN)	*Far Eastern tick-borne encephalitis* [147]	
	Togaviridae	*Eastern equine encephalitis virus*		NC_003899
		Venezuelan equine encephalitis virus		NC_001449
Toxin (protein)	Bacteria, Low G+C gram positive, Clostridia	*Botulinum toxin*		AF488749 AB088207 AB082519
		Clostridium perfringens epsilon toxin		M95206 M80837
	Bacteria, Low G+C gram positive, Bacilli	*Staphylococcal enterotoxin B*		M11118
	Bacteria, gammaproteobactera	*Shigatoxin*		AB035142 AB035143 AF461169 AF461170
	Animal, mollusca, gastropoda	*Conotoxins*		
	Plant, embryophyta	*Abrin*		
		Ricin		
Toxin (small molecule)	Bacteria, gammaproteobacteria	*Tetrodotoxin*		
	Protists, alveolata	*Saxitoxin*		
	Fungi, ascomycota	*Diacetoxyscirpenol*		
		T-2 toxin		
