# Supplementary material for: The Microbial Rosetta Stone Database: A compilation of global and emerging infectious microorganisms and bioterrorist threat agents
Source: BMC Microbiol. 2005 Apr 25;5:19. doi: 10.1186/1471-2180-5-19 (PMC1127111; doi:10.1186/1471-2180-5-19)
Supplement: Additional File 10 — USDA high consequence pathogens [PDF]. Literature used in population of the table included: [146,148]. [file 1471-2180-5-19-S10.pdf]

# Additional File 10.  USDA High Consequence Pathogens

| *Phylogeny* | | *NCBI Name* | Threat List Name or Synonym | Host | Accession |
|---|---|---|---|---|---|
| Prion | | *Bovine spongiform encephalopathy prion* | *Bovine spongiform encephalopathy agent* | A | S55629 |
| Fungi | Ascomycota | *Coccidioides immitis* | | A | |
| | Basidiomycota | *Phakopsora pachyrhizi* | | P | |
| | Chytridiomycota | ----- | *Synchytrium endobioticum* | P | |
| Eukaryota | Oomycetes | ----- | *Peronosclerospora philippinensis* | P | |
| | | ----- | *Sclerophthora rayssiae var. zeae* | P | |
| Bacteria | Alphaproteobacteria | *Brucella melitensis* | | A | NC_003317 NC_003318 |
| | | *Brucella melitensis biovar Abortus* | *Brucella abortus* | A | |
| | | *Brucella melitensis biovar Suis* | *Brucella suis* | A | NC_004310 NC_004311 |
| | | *Candidatus Liberibacter africanus* | *Liberobacter africanus* | P | |
| | | *Candidatus Liberibacter asiaticus* | *Liberobacter asiaticus* | P | |
| | | *Ehrlichia ruminantium* | *Cowdria ruminantium* | A | |
| | Betaproteobacteria | *Burkholderia mallei* | | A | NC_002970* |
| | | *Burkholderia pseudomallei* | | A | NC_002930* http://www.sanger.ac.uk/Projects/B_pseudomallei/ |
| | | *Ralstonia solanacearum* | (race 3, biovar 2) | P | |
| | Gammaproteobacteria | *Coxiella burnetii* | | A | NC_002971 |
| | | *Francisella tularensis* | | A | |
| | | *Xanthomonas oryzae pv. oryzicola* | | P | |
| | | *Xylella fastidiosa* | (citrus variegated chlorosis strain) | P | NC_002488 NC_004556 |
| | Firmicutes | *Bacillus anthracis* | | A | NC_003997 NC_003995* NC_004352* NC_002925* NC_004126* |
| | | *Clostridium botulinum* | Botulinum neurotoxin producing species of *Clostridium* [146] | A | NC_003223* http://www.sanger.ac.uk/Projects/C_botulinum/ |
| | | *Clostridium baratii* | Botulinum neurotoxin producing species of *Clostridium* [146] | A | |
| | | *Clostridium butyricum* | Botulinum neurotoxin producing species of *Clostridium* [146] | A | |
| | | *Mycoplasma capricolum subsp. capripneumoniae* | | A | |
| | | *Mycoplasma mycoides* | | A | |
| DNA Virus | *Asfarviridae* | *African swine fever virus* | | A | NC_001659 |
| | *Herpesviridae* | *Alcelaphine herpesvirus 1* | Malignant catarrhal fever virus (exotic) | A | NC_002531 |
| | *Poxviridae* | *Camelpox virus* | | A | NC_003391 |
| | | *Goatpox virus* | | A | NC_004003 |
| | | *Lumpy skin disease virus* | | A | NC_003027 |
| | | *Sheeppox virus* | | A | NC_004002 |
| - Strand RNA Virus | *Bunyaviridae* | *Akabane virus* | | A | |
| | | *Rift Valley fever virus* | | A | NC_002043 NC_002044 NC_002045 |
| | *Orthomyxoviridae* | *Avian influenza virus* | (highly pathogenic) (H5N1) | A | |
| | *Paramyxoviridae* | *Hendra virus* | | A | NC_001906 |
| | | *Menangle virus* | | A | |
| | | *Newcastle disease virus* | *Newcastle disease virus (VVND)* | A | |
| | | *Nipah virus* | | A | NC_002728 |
| | | *Peste-des-petits-ruminants virus* | | A | |
| | | *Rinderpest virus* | | A | |
| | *Rhabdoviridae* | *Vesicular stomatitis virus* | (exotic) | A | NC_001560 |
| + Strand RNA Virus | *Flaviviridae* | *Classical swine fever virus* | | A | NC_002657 |
| | | *Japanese encephalitis virus* | | A | NC_001437 |
| | *Picornaviridae* | *Foot-and-mouth disease virus* | | A | NC_002554 NC_003992 NC_004004 NC_004915 |
| | | *Swine vesicular disease virus* | | A | |
| | *Potyviridae* | *Plum pox virus* | | P | NC_001445 |
| | *Togaviridae* | *Eastern equine encephalitis virus* | | A | NC_003899 |
| | | *Venezuelan equine encephalitis virus* | | A | NC_001449 |
| dsRNA Virus | *Reoviridae* | *African horse sickness virus* | | A | |
| | | *Bluetongue virus* | (exotic) | A | |
| Toxin (protein) | Bacteria, Low G+C gram positive, Clostridia | *Botulinum toxin* | | A | AF488749 AB088207 AB082519 |
| | | *Clostridium perfringens epsilon toxin* | | A | M95206 M80837 |
| | Bacteria, Low G+C gram positive, Bacilli | *Staphylococcal enterotoxin B* | | A | M11118 |
| | Bacteria, gammaproteobactera | *Shigatoxin* | | A | AB035142 AB035143 AF461169 AF461170 |
| Toxin (small molecule) | Fungi, ascomycota | *T-2 toxin* | | A | |
